# Impact of Magnetic Field on Dose Distribution in MR-Guided Radiotherapy of Head and Neck Cancer

**DOI:** 10.3389/fonc.2020.01739

**Published:** 2020-09-08

**Authors:** Wenlong Xia, Ke Zhang, Minghui Li, Yuan Tian, Kuo Men, Jingbo Wang, Junlin Yi, Yexiong Li, Jianrong Dai

**Affiliations:** Department of Radiation Oncology, National Cancer Center/National Clinical Research Center for Cancer/Cancer Hospital, Chinese Academy of Medical Sciences and Peking Union Medical College, Beijing, China

**Keywords:** MR-guided radiotherapy, head and neck cancer, MR-linac, electron return effect, plan quality metric

## Abstract

**Purpose:**

This study investigates the impact of the magnetic field on plan quality and dose at the tissue–air interface in MR-guided radiotherapy of head and neck cancer.

**Materials and Methods:**

The charts of 10 patients with hypopharyngeal carcinoma who were treated with conventional fractionated radiotherapy were collected and reviewed. The skin and tissues containing air cavities were contoured. Three plans using 9 fields of intensity-modulated radiation therapy were generated for each patient in the Monaco treatment planning system of an Elekta Unity MR-linac. The first plan was optimized without the magnetic field (plan_0T_). The second plan was recalculated in the presence of a 1.5-T magnetic field (plan_1.5T_reCal_) using the same segment shape and monitor units as the first plan. The third plan was reoptimized in the presence of a 1.5-T magnetic field (plan_1.5T_reOpt_) using the same cost function as the first plan. The dose to the skin and tissues containing air cavities were compared across the three types of plans. A plan-quality metric method was used to evaluate the plan quality according to the clinical requirements.

**Results:**

The skin dose was increased in the presence of the 1.5-T magnetic field, and the amplitude increase of plan_1.5T_reOpt_ (ΔD_mean_ 1.30 ± 0.42 Gy, ΔD_max_ 1.68 ± 1.36 Gy) was smaller than that of plan_1.5T_reCal_ (ΔD_mean_ 1.81 ± 0.79 Gy, ΔD_max_ 5.43 ± 2.26 Gy). There were no significant differences in terms of the metrics of interfaces of tissues containing air cavities except for an increased maximum dose to the larynx and trachea. The plan quality of plan_1.5T_reCal_ (68.0 ± 9.2) was significantly worse than that of plan_0T_ (82.2 ± 7.0), and the plan quality of plan_1.5T_reOpt_ (80.0 ± 7.0) was similar to that of plan_0T_.

**Conclusion:**

The presence of a 1.5-T magnetic field had an apparent impact on the dose distribution, in particular, a significant increase in the skin dose. The plan quality of plan_1.5T_reOpt_ was similar to that of the original plan_0T_ when the same cost function was used.

## Introduction

Magnetic resonance (MR) provides better image contrast than computed tomography (CT), especially for soft tissue, and it does not expose the patient to ionizing radiation (1, 2). To implement MR image guidance during radiation treatment, the integration of MR imaging with a linear accelerator (MR-linac) has been developed, covering both low and high magnetic field strength (3–6). In MR-linac, the Lorentz force exerted by the static magnetic field causes secondary electrons to move perpendicularly to their velocity direction. Inside the body, the Lorentz force reduces the buildup depth and causes asymmetry in the lateral beam profile (7). At the tissue–air interface, the magnetic field returns the electrons that have exited the tissue to the surface, which is called the electron return effect (ERE) (8). This effect is obvious at interfaces between layers with great density differences and results in significant dose variation at such interfaces.

In radiotherapy of head and neck (H&N) cancer, MR image guidance provides many benefits (9), such as delineation of target volumes (10), motion management (11), and adaption of anatomical changes (12), which are useful for real-time, high-contrast visualization of the tumor and organs at risk (OARs). However, apart from the benefits of MR image guidance, it is important to investigate the impact of the magnetic field on dose distribution. A single beam at a highly oblique surface can result in a dose increase of up to 56% to the exit point (13). The ERE can vary in terms of the incident angle of the beam to the surface, and oblique angles induce the largest dose increase at the interface. However, at perpendicular interfaces, this effect can be compensated by using opposing beams (8, 13). Most parts of target volumes in H&N are superficial and relatively large, which results in a relatively large area of skin irradiation. Furthermore, H&N tissues contain *in vivo* air cavities, including the nasopharynx, oropharynx, larynx, and trachea, in which the ERE may be problematic because the tissue–air interface affects the dose distribution.

In recent years, with the development of clinical applications of MR-linac, there has been increasing interest in the impact of the magnetic field on dose distribution, and a few studies have been presented. Chuter et al., assessed the robustness of MR-linac radiotherapy in the case of anatomical changes in H&N cancer, indicating no significant differences between the doses in the primary target volume (PTV) and OARs related to weight loss with or without a magnetic field (14). Heijst et al., investigated the effects of a magnetic field on the skin dose in breast radiotherapy, finding that accelerated partial breast irradiation induced less of an increase to the skin dose than whole breast irradiation (15). Bol et al., conducted a comprehensive simulation using a phantom to investigate static and moving air cavities within the target area. They found intrinsic ERE compensation as a result of using equidistant and opposing beams, and they observed that additional compensation can be provided in intensity-modulated radiation therapy (IMRT) in the case of correct positioning of the air cavity (16). Raaijmakers et al., studied IMRT plans in the presence of a 1.5-T magnetic field for three cases with different target sites (prostate cancer, laryngeal cancer, and oropharyngeal cancer). The results show that the magnetic field induced a minimal difference of dose distribution and an apparent increment of skin dose (17). However, for tumors located in the H&N, in which large skin areas and many *in vivo* air cavities are irradiated, no report has presented a comprehensive statistical comparison of the IMRT plans with and without a magnetic field. It is necessary to investigate the statistical differences between 0 T and 1.5 T in terms of the dosimetric parameters of IMRT plans and the doses to skin and tissues containing air cavities.

Here, we present the first comprehensive study investigating the impact of the magnetic field on both plan quality and dose variation at the tissue–air interface in MR-guided radiotherapy of H&N cancer. First, in order to conduct a quantitative evaluation of the ERE-induced dose increment in the tissue–air interfaces, the skin and the interfaces of tissues containing air cavities were specifically contoured. Then, in order to evaluate overall treatment plan quality, a plan-quality metric (PQM) scoring procedure was introduced according to the clinical requirements. Moreover, we generated three types of IMRT plans for each case: the original plan without the magnetic field (plan_0T_), a recalculated plan in the presence of 1.5 T (plan_1.5T_reCal_), and a reoptimized plan in the presence of 1.5 T (plan_1.5T_reOpt_). We included plan_1.5T_reCal_ to help us to determine the quantitative differences to the IMRT plans caused by only the addition of a 1.5-T magnetic field. Finally, we conducted statistical analysis to compare the dose parameters among the three types of plans using data from 10 patients with hypopharyngeal carcinoma. Therefore, this study adds four main contributions to the existing literature: (1) evaluation of the ERE-induced dose increment at specifically contoured tissue–air interfaces, (2) assessment of overall plan quality based on a PQM scoring procedure, (3) design of three types of plans for each case, and (4) statistical analysis of the data from 10 patients with hypopharyngeal carcinoma.

Currently, an Elekta Unity MR-linac (1.5-T magnetic field, fixed isocenter, non-rotating collimator) is installed and commissioned in our center. We used a 9-field (9F) IMRT technique in the MR-linac to generate three types of plans for each of the 10 patients with hypopharyngeal carcinoma. Based on comparisons among the three types of plans, we aimed to investigate the impact of a magnetic field on dose distribution in MR-guided radiotherapy of H&N cancer, concentrating on (1) magnetic field–induced variation of plan quality and (2) ERE-induced dose increases to the skin and tissues containing air cavities.

## Materials and Methods

### Patient Selection and Regions of Interest

We reviewed the data of 10 patients with hypopharyngeal carcinoma who were treated with conventional fractionated radiotherapy. The patients’ median age was 59 years (range: 40–66 years). The patients’ tumor staging and dose prescriptions are listed in Table 1. Because the maximum field size of the Unity MR-linac is 57 cm × 22 cm, the length of PTV for each patient was shorter than 20 cm.

**TABLE 1:** Tumor staging and prescriptions.

	Number of patients
**Tumor staging**	
T4	4 (N2 = 3/N3 = 1)
T2	4 (N0 = 1/N2 = 3)
T1	2 (N2 = 1/N3 = 1)
**Prescribed dose/fractionation**	
PGTVtb	69.96Gy/2.12Gy/33f
GTVnd	69.96Gy/2.12Gy/33f
PTV	60.06Gy/1.82Gy/33f

**FIGURE 1:**
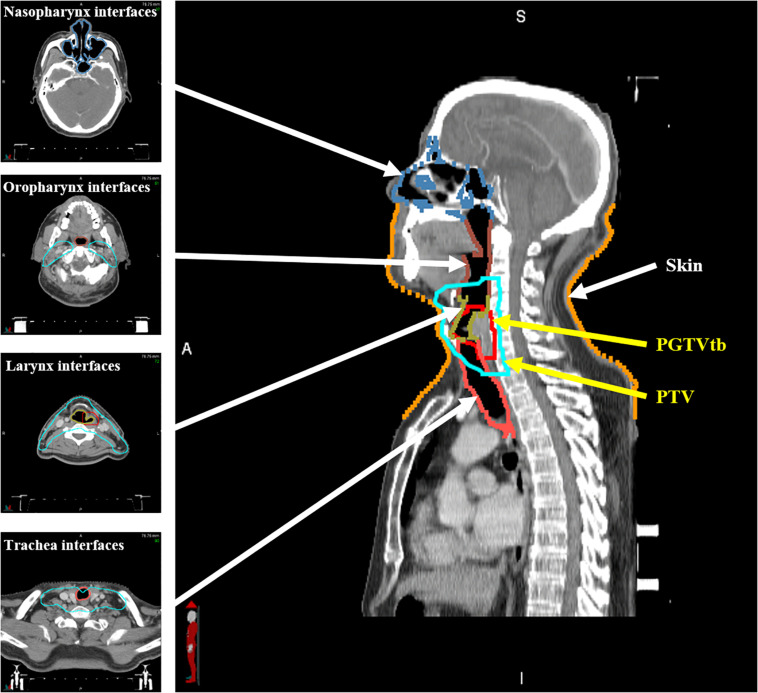
A patient CT scan with delineated regions of interest.

The patients underwent CT scans in a supine position with 3-mm slice thickness and 1 mm × 1 mm planar voxel size. Gross tumor volume in the tumor bed (GTVtb), planning gross tumor volume in the tumor bed (PGTVtb), the involved lymph nodes (GTVnd), the clinical target volume, and the planning target volume (PTV) were contoured by senior physicians. Relevant OARs, including the brain stem, spinal cord, parotids, larynx, trachea, and thyroid gland were also delineated. Extra 3- and 5-mm margins were added to the brain stem and spinal cord, respectively, as the planning OAR volume (PRV). Furthermore, as shown in Figure 1, in order to quantitatively evaluate the ERE-induced dose increment in the tissue–air interfaces, the skin was contoured to include the first 1 mm under the outline of the body, and the interfaces of tissues containing air cavities (nasopharynx, oropharynx, larynx, and trachea) were contoured to include the first 1 mm outside of the air cavities.

### IMRT Planning

Treatment planning was performed using the Monaco (v5.40.01, Elekta AB, Stockholm, Sweden) treatment planning system (TPS). As described by (18), the GPU Monte Carlo dose (GPUMCD) algorithm in the Monaco TPS allows the inclusion of a 1.5-T magnetic field. The algorithm has shown good consistency with GEANT4 calculations at both 0 T and 1.5 T (19). A step-and-shoot IMRT dose-delivery technique is used in the Unity MR-linac because the volumetric modulated arc therapy dose-delivery technique is not yet available.

Considering the location of the PTV and its feature of symmetric distribution, a 9F IMRT plan is generally used in our clinical practice. For each patient, three 9F IMRT plans were generated in the Monaco TPS in the presence of 0-T and 1.5-T magnetic fields with the following parameters: the minimum segment area was set to 4 cm^2^, the minimum segment width was set to 0.5 cm, low fluence smoothing, minimum monitor unit (MU) per segment was set to 4 MU, the maximum number of segments was set to 150, and the statistical uncertainty per calculation was set to 1%. The first plan (plan_0T_) was generated at 0 T using a set of optimization objectives from a previously stored template for the optimization of hypopharyngeal carcinoma treatment plans. The second plan (plan_1.5T_reCal_) was directly recalculated under a 1.5-T magnetic field using the same segment shape and monitor units as the first plan. The third plan (plan_1.5T_reOpt_) was reoptimized under a 1.5-T magnetic field using the same cost function as the first plan. The optimization process of plan_1.5T_reOpt_ was as follows: After plan_0T_ was completed and saved, we reset the calculation engine (remove fluence, segments, and dose), changed the beam type from “MRLNoMag” to “MRL1.5T,” and optimized the plan using the same cost function as plan_0T_. On the basis of plan_0T_, the optimization time of plan_1.5T_reOpt_ was about 10 min. During the optimization process, there was just one button click “Batch Optimization” to start optimization and batch through optimization and sequencing stages. The plans were optimized based on the fully optimized fluence maps with a 3-mm grid resolution. After the optimization was finished, the dose was recalculated with a 2-mm grid resolution.

To address the effects of variation stemming from the optimization and Monte Carlo (MC) dose calculation, the above planning process was repeated for each plan. Therefore, two sets of data were collected for each plan, and averaged values were calculated for dosimetric evaluation.

### Study Endpoints

To compare the three types of plans, dose-volume histograms were calculated for all structures and corresponding evaluation parameters.

The dose parameters of the targets (PGTVtb, GTVnd, and PTV) included the following metrics: (i) V*_*p*_* (the percentage of the target volume receiving the prescribed dose), which describes target coverage. (ii) The homogeneity index (HI) (20), defined as:

(1) $$H\,I=100\%\times \frac{D\,2\%-D\,98\%}{D\,50\%}$$

D2, D98, and D50% are the minimum doses delivered to 2, 98, and 50% of the PTV, respectively. The closer the HI value is to 0, the better the homogeneity. (iii) The conformity index (CI) (21), defined as:

(2) $$C\,I=\frac{T\,V_{P\,T\,V}^{2}}{V_{P\,T\,V}\times T\,V}$$

V_PTV_ is the volume of the target, and TV_PTV_ is the portion of the V_PTV_ within the prescribed isodose line. TV is the treated volume of the prescribed isodose line. The closer the CI value is to 1, the better the conformity.

Several parameters were compared for the OARs: (i) the maximum dose (D_max_) to the brain stem and brain stem PRV; (ii) the maximum dose (D_max_) to the spinal cord and spinal cord PRV; (iii) the mean dose to the parotids; (iv) the mean dose to normal tissue (NT), defined as the volume inside the body and greater than 1 cm from the PTV. The larynx, trachea, and thyroid gland were not involved because they were entirely or partially inside the targets. Moreover, the maximum and mean doses were compared for skin and the interfaces of tissues containing air cavities.

### Plan Quality Metrics

**FIGURE 2:**
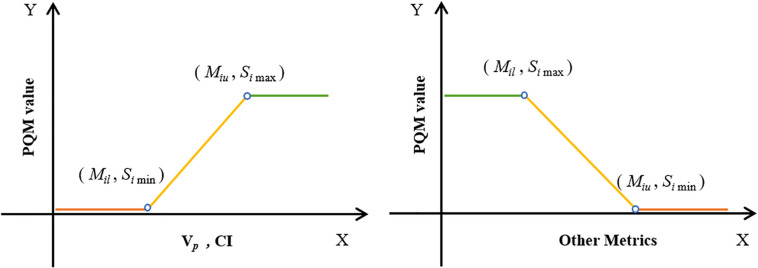
Schematic plots of PQM value functions for 15 submetrics (see Table 2).

According to the concept of PQM proposed by Benjamin (22) and the plan-quality score S_D_ proposed by Bohsung (23), a new PQM scoring procedure for treatment plans with 15 related submetrics was defined. Each metric has a unique quantity and PQM value function that is used to calculate a point value, and the ranges of the corresponding PQM values were uniformly set from 0 to 10. A description of each PQM metric is shown in Table 2. Figure 2 presents the schematic plots of the PQM value functions. The quality score S of each plan is the sum of the PQM values of the 15 submetrics, defined as follows:

**TABLE 2:** Evaluation interval of metric parameters along with their point value range.

Structure	Metric		
	
	Parameter	Lower limit	Upper limit
PGTVtb	V_69.96_ (%)	80	95
	CI	0	1
	HI	0	0.2
GTVnd	V_69.96_ (%)	80	95
	CI	0	1
	HI	0	0.2
PTV	V_60.06_ (%)	90	95
	CI	0.6	1
	HI	0	0.3
Brain stem	D_max_ (Gy)	25	40
Brain stem PRV	D_max_ (Gy)	30	45
Spinal cord	D_max_ (Gy)	30	40
Spinal cord PRV	D_max_ (Gy)	35	45
Parotids	D_mean_ (Gy)	40	55
NT	D_mean_ (Gy)	10	30

(3) $$S=\sum_{i=1}^{k}S_{i}$$

(4) $$S_{i}=\left\{ \begin{array}{cc} \frac{M_{i}-M_{i\,l}}{M_{i\,u}-M_{i\,l}}\times \left(S_{i\,\max}-S_{i\,\min}\right), & V_{p}\,\text{ and CI}\\ \frac{M_{i\,u}-M_{i}}{M_{i\,u}-M_{i\,l}}\times \left(S_{i\,\max}-S_{i\,\min}\right), & \text{else} \end{array}\right.$$

where *k* is the number of submetrics, *S*_*i*_ is the PQM value of the corresponding metric (*M*_*i*_), and *M*_*il*_ and *M*_*iu*_ are the lower and upper limits of *M*_*i*_, respectively. *S*_*imax*_ and *S*_*imin*_ are the maximum and minimum PQM values of *M*_*i*_.

The interval of each metric was determined according to both clinical requirements and the minimum (or maximum) value of the corresponding metric’s recorded data. For instance, the clinical requirement of V_69.96_ was 95% and the minimum recorded V_69.96_ data point across all plans was 81.9%. Therefore, the interval was set to range from 80 to 95%. In this way, all plans in this control experiment could be evaluated using the PQM scoring procedure.

### Statistical Analysis

The statistical significance of differences between all dosimetric parameters was tested by performing the Wilcoxon signed rank test in SPSS v17 (IBM Corp.). Values of *p* < 0.05 were considered to represent statistically significant results.

## Results

### Effect of 1.5-T Magnetic Field on Plan Quality

**FIGURE 3:**
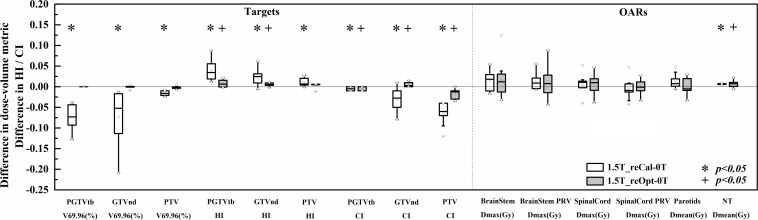
Differences in the investigated dose–volume metrics between the plans designed for the 1.5-T MR-linac as either recalculated or reoptimized and the 0 T linac. Numerically positive differences mark an increase in the respective metric for the 1.5-T MR-linac plans. Displayed are the first and third quartiles (boxes), medians (bands inside), average values (crosses), standard deviations (whiskers), and outliers (circles).

The differences in dose–volume metrics between the treatment plans designed for 1.5 T and 0 T are presented in Figure 3. Statistically significant differences were found in the metrics involving dose coverage, HI, CI, and NT.

With regard to PGTVtb, GTVnd, and PTV, all metrics of the recalculated plans were significantly worse in the presence of a 1.5-T magnetic field (*p* < 0.05), including decreased dose coverage V*_*p*_* (−0.071 ± 0.027, −0.072 ± 0.059, and −0.016 ± 0.005, respectively), increased HI (0.039 ± 0.023, 0.023 ± 0.020, and 0.011 ± 0.010, respectively), and decreased CI (−0.005 ± 0.005, −0.032 ± 0.027, and −0.065 ± 0.025, respectively). Some metrics of plan_1.5T_reOpt_ also got worse significantly in the presence of the 1.5-T magnetic field (*p* < 0.05), including increased HI in PGTVtb and GTVnd (0.008 ± 0.009 and 0.005 ± 0.004, respectively), decreased CI in PGTVtb and PTV (−0.004 ± 0.005 and −0.017 ± 0.011, respectively), and increased CI in GTVnd (0.005 ± 0.005). However, for all metrics in the targets section, the magnitudes of the differences in the reoptimized plans were smaller than those in the recalculated plans. Most metrics of the OARs increased in the presence of a 1.5-T magnetic field for both the recalculated and reoptimized plans. However, no significant differences were found except for an increased mean NT dose in the recalculated and reoptimized plans (0.13 ± 0.02 Gy and 0.12 ± 0.14 Gy, respectively).

Table 3 shows the plan quality scores of the three types of plans. The plan quality score indicates the treatment plan’s overall quality. The quality of the recalculated plan was significantly decreased in the presence of a 1.5-T magnetic field (plan_1.5T_reCal_ – plan_0T_: −14.2 ± 5.4, *p* < 0.05). However, the reoptimized plan significantly improved the plan quality (plan_1.5T_reOpt_ – plan_1.5T_reCal_: 12.0 ± 4.7, *p* < 0.05). There was no statistically significant difference between plan_0T_ and plan_1.5T_reOpt_. According to the plan-quality scores, plan_1.5T_reOpt_ had equivalent quality to plan_0T_.

**TABLE 3:** The quality scores of the three types of plans.

Patient number	0 T	1.5T_reCal	1.5T_reOpt
1	88.4	72.8	81.6
2	73.0	58.1	67.8
3	89.7	81.6	90.0
4	82.0	65.6	81.3
5	79.7	75.9	79.8
6	79.2	62.1	76.4
7	88.8	64.9	82.2
8	72.8	52.2	74.1
9	93.0	81.7	92.6
10	75.8	65.7	74.7
Mean	82.2 ± 7.0	68.0 ± 9.2	80.0 ± 7.0
Median	80.9	65.6	80.6

Significance	0T vs. 1.5T_reCal	0T vs. 1.5T_reOpt	1.5T_reCal vs. 1.5T_reOpt
level	0.005	0.059	0.005

### Effects of 1.5-T Magnetic Field on Dose Distribution to Skin and Tissues Containing Air Cavities

**FIGURE 4:**
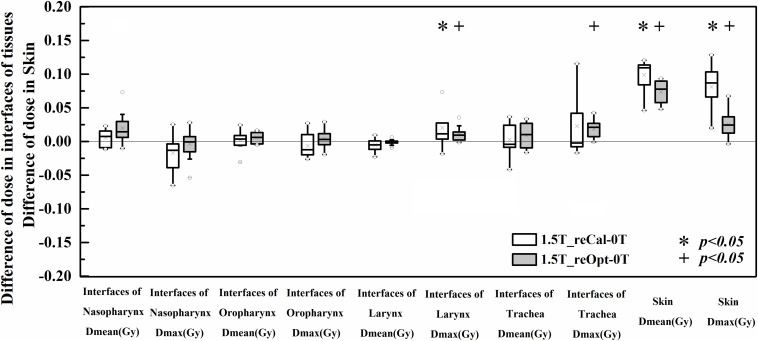
Dose differences in the interfaces of tissues containing air cavities and skin between the plans designed for the 1.5-T MR-linac as either recalculated or reoptimized and the 0 T linac. Numerically positive differences mark an increase in the respective metric for the 1.5-T MR-linac plans. Displayed are the first and third quartiles (boxes), medians (bands inside), average values (crosses), standard deviations (whiskers), and outliers (circles).

The differences of dose deposition to skin and tissues containing air cavities between the treatment plans designed in the presence of 1.5 T and 0 T are shown in Figure 4. No statistically significant differences in metrics were found at the interfaces of tissues containing air cavities except for an increased maximum dose to the larynx (plan_1.5T_reCal_ – plan_0T_: 1.58 ± 2.30 Gy, plan_1.5T_reOpt_ – plan_0T_: 0.85 ± 0.91 Gy) and increased maximum dose to the trachea (plan_1.5T_reOpt_ – plan_0T_: 1.34 ± 0.91 Gy). However, all metrics showed significant differences in the skin (*p* < 0.05), including increased mean dose (plan_1.5T_reCal_ – plan_0T_: 1.81 ± 0.79 Gy, plan_1.5T_reOpt_ – plan_0T_: 1.30 ± 0.42 Gy) and increased maximum dose (plan_1.5T_reCal_ – plan_0T_: 5.43 ± 2.26 Gy, plan_1.5T_reOpt_ – plan_0T_: 1.68 ± 1.36 Gy). The skin dose increment in plan_1.5T_reOpt_ was smaller than that in plan_1.5T_reCal_ (*p* < 0.05).

**FIGURE 5:**
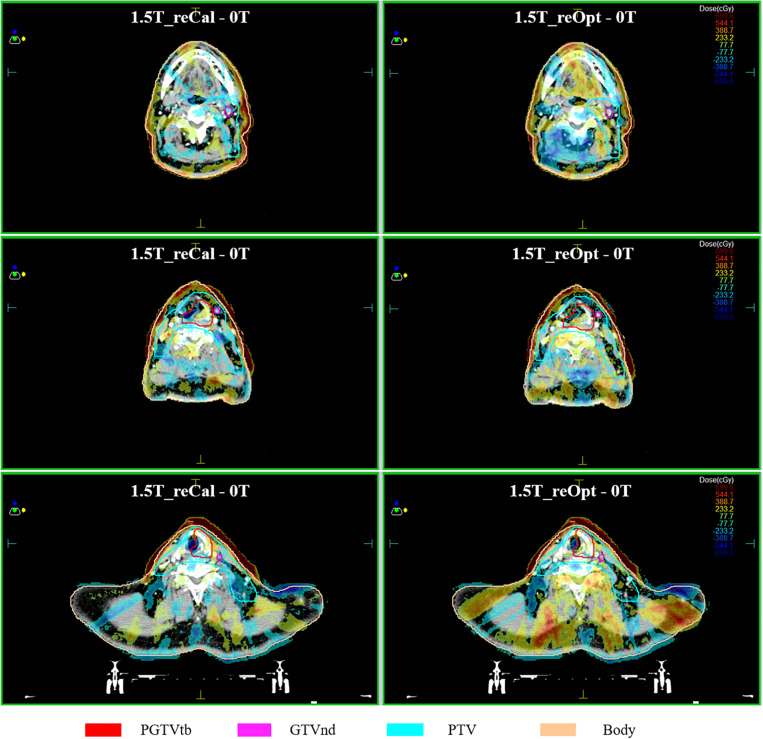
Maps of dose differences (in cGy) per voxel relative to the situation of no magnetic field. Examples of three transversal slices are depicted in each consecutive row, and two types of plans are arranged per column, i.e., left: 1.5 T_reCal – 0T; Right 1.5 T_reOpt – 0T. Differences range from −700 cGy (dark blue) to +700 cGy (dark red).

The voxel-wise dose-difference maps relative to plan_0T_ are shown in Figure 5. For both types of plans, the highest dose increases were observed in the most superficial layer of the skin close to the targets, and the dose difference gradually decreased from the skin toward the inside of the body. In some slices at interfaces of the tissues containing air cavities, we observed that the dose decreased on one side and increased on the other side. Because the segment shape and monitor units of the reoptimized plan changed, the overall dose difference of plan_1.5T_reOpt_ (right column) was greater than that of plan_1.5T_reCal_ (left column). Around the body contour, part of the dose was distributed outside the body. The reason may be that the MC dose calculation takes into account the air outside the body. According to the MC calculated dose distribution shown in Figure 2D of (24), there was dose distribution in the air outside of the body. However, for a treatment plan, we usually do not consider the dose distribution outside the body.

## Discussion

In this study, we investigated the impacts of a 1.5-T magnetic field on IMRT plans for H&N cancer, which mainly include two parts: the variation of plan quality and ERE-induced dose increases to the skin and interfaces of tissues containing air cavities. Three types of plans were designed: one original plan at 0 T (plan_0T_) and two plans in the presence of a 1.5-T magnetic field (plan_1.5T_reCal_ and plan_1.5T_reOpt_).

In the first part, we observed that the metrics of the targets were significantly worse in plan_1.5T_reCal_ than in plan_0T_, especially for the targets with small volume (GTVnd). After the plan was reoptimized in the presence of a 1.5-T magnetic field (plan_1.5T_reOpt_), the target metrics were close to those in plan_0T_. Relative to the target volumes, the OARs were less affected by the magnetic field in both plan_1.5T_reCal_ and plan_1.5T_reOpt_. The results indicate that the target volumes were less robust to the presence of the magnetic field than OARs in H&N cancer treatment plans. Theoretically, the magnetic field–induced changes are caused by the variation of dose deposition in tissues, in which the trajectories of secondary electrons are affected by the magnetic field (25, 26). The pencil beam dose deposition kernel can become clearly asymmetric in the direction perpendicular to the 1.5-T magnetic field (7).

The quantitative plan-quality scoring procedure introduced in this study considers all metrics for both targets and OARs. There are several ways to assign various scoring proportions to targets and OARs by using different weights, different numbers of metrics for each structure or both. In our study, we simply regarded all evaluated metrics as equally important and used different numbers of metrics to assign the scoring proportions of targets and OARs. Different methods could lead to different scores. However, with an appropriate assignment of the scoring proportions, different methods may not affect the comparison result. The selection should be determined by both senior physicians and experienced planners. It should be noted that the subjective nature of defining the scale and weights of the individual components of the PQM is a shortcoming of the metric and cannot be avoided. Despite this, PQM can be an important index for quantitative evaluation of a treatment plan’s overall quality. Compared with that of plan_0T_, the plan quality score of plan_1.5T_reCal_ was significantly decreased and that of plan_1.5T_reOpt_ was close (slightly decreased but no significant difference). This result is in accordance with recent studies of different types of tumors, which report that reoptimized MR-linac plans in the presence of a 1.5-T magnetic field are clinically equivalent to clinical plans generated using conventional linac systems (27–29). Unlike those studies, our study further analyzed plan_1.5T_reCal_ using the same segment shape as that used for plan_0T_. This helps us to understand the quantitative impacts on plan quality only considering the presence of a 1.5-T magnetic field, that is, the robustness of conventional IMRT plans to the presence of a magnetic field. We observed that plan_1.5T_reCal_ was substantially inferior to plan_0T_, and the results of the former were not clinically acceptable. However, plan_1.5T_reOpt_ achieved similar quality as plan_0T_.

In the second part of this work, we investigated the ERE-induced dose increases to the skin and tissues containing air cavities. Irradiation of the skin and *in vivo* mucous membranes is an unavoidable but transient side effect of curative radiotherapy of H&N cancer, and it may result in mucositis and ulceration (30). The observed dose increase to the skin in the MR-linac plans was as expected from previous studies (15, 27, 31, 32). In terms of both the mean and maximum doses to the skin, the magnitude increase of plan_1.5T_reOpt_ was smaller than that of plan_1.5T_reCal_. This is because the use of the NT constraint in the reoptimized process achieved better skin sparing. No significant differences in metrics were observed in the interfaces of tissues containing air cavities except for the increased maximum dose observed in the larynx and trachea, which were partially or fully located within the target volumes. As described by (16, 17), the influence of the magnetic field (particularly the ERE) can be compensated by using an opposing beam. Thus, the 9F IMRT (which is evenly distributed throughout all 360°) adopted in this study partially compensates for the ERE-induced dose variation at tissue–air interfaces, which may result in the lack of significant changes to the mean dose. The dose differences on both sides of the tissue–air interface can be observed in Figure 5, which shows a dose decrease on one side and a corresponding dose increase on the other side. These changes are likely caused by the irregular shapes of different segments and the different locations of the *in vivo* air cavities, which may particularly increase the maximum dose to the larynx and trachea.

In this study, we mainly investigated the impacts of the magnetic field on IMRT plans, and we used the same cost function for all plans to prevent other variables from affecting the results. However, the cost function that would yield the best plans with and without the magnetic field could be different. Therefore, through the appropriate modification of cost function, the impacts of the magnetic field (e.g., increased dose to skin and tissues containing air cavities) can be further reduced.

## Conclusion

In summary, this study shows that the magnetic field has a great impact on the quality of plan_1.5T_reCal_, the recalculated plan. However, reoptimizing the plan in the presence of a 1.5-T magnetic field (plan_1.5T_reOpt_) increases the feasibility of achieving a clinically acceptable treatment plan for hypopharyngeal carcinoma. Furthermore, there is a significant increase to the skin dose in the presence of a 1.5 T magnetic field, and there were no significant differences in the metrics of interfaces of tissues containing air cavities except for an increased maximum dose to the larynx and trachea.

## Data Availability Statement

The datasets presented in this article are not readily available because the datasets generated during the current study are not publicly available due to data security requirement of our hospital. Requests to access the datasets should be directed to WX, erikxia10@163.com.

## Ethics Statement

This study was carried out in accordance with the Declaration of Helsinki and approved with exemption from informed consent by the independent Ethics Committee of Cancer Hospital, Chinese Academy of Medical Sciences. Written informed consent for participation was not required for this study in accordance with the National Legislation and the Institutional Requirements.

## Author Contributions

WX wrote the programs, performed data analysis, and drafted the manuscript. KZ, ML, YT, KM, and JW helped to collect and analyze the patients’ data. JY, YL, and JD guided the study and participated in discussions and preparation of the manuscript. All authors discussed and conceived of the study design, read, discussed, and approved the final manuscript.

## Conflict of Interest

The authors declare that the research was conducted in the absence of any commercial or financial relationships that could be construed as a potential conflict of interest.
