# Corrigendum: Impact of Magnetic Field on Dose Distribution in MR-Guided Radiotherapy of Head and Neck Cancer

**DOI:** 10.3389/fonc.2020.604231

**Published:** 2020-11-13

**Authors:** Wenlong Xia, Ke Zhang, Minghui Li, Yuan Tian, Kuo Men, Jingbo Wang, Junlin Yi, Yexiong Li, Jianrong Dai

**Affiliations:** Department of Radiation Oncology, National Cancer Center/National Clinical Research Center for Cancer/Cancer Hospital, Chinese Academy of Medical Sciences and Peking Union Medical College, Beijing, China

**Keywords:** MR-guided radiotherapy, head and neck cancer, MR-linac, electron return effect, plan quality metric

In the published article, there was an error in the authors' affiliation. Instead of “National Cancer Center, National Clinical Research Center for Cancer, Cancer Hospital, Chinese Academy of Medical Sciences, Peking Union Medical College, Beijing, China,” it should be “Department of Radiation Oncology, National Cancer Center/National Clinical Research Center for Cancer/Cancer Hospital, Chinese Academy of Medical Sciences and Peking Union Medical College, Beijing, China.”

The authors apologize for this error and state that this does not change the scientific conclusions of the article in any way. The original article has been updated.

